# Uncloggable ventriculoperitoneal shunt system for hydrocephalus via an integrated soft robotic device: CLEARS device

**DOI:** 10.1007/s10544-025-00769-8

**Published:** 2025-09-11

**Authors:** Yau C. Yun, David R. Santiago-Dieppa, Minghao Li, Aditya Vasan, Alexander Khalessi, James Friend

**Affiliations:** 1 Medically Advanced Devices Laboratory, Center for Medical Devices and Biomechanics, Department of Mechanical and Aerospace Engineering, Jacobs School of Engineering and Department of Surgery, School of Medicine, University of California San Diego, 9500 Gilman Drive, La Jolla, CA 92093-0411 USA; 2 Department of Neurosurgery, School of Medicine, University of California San Diego, 9500 Gilman Drive, La Jolla, CA 92093 USA; 3 Materials Science and Engineering Program, Jacobs School of Engineering, University of California San Diego, 9500 Gilman Drive, La Jolla, CA 92093 USA

**Keywords:** Shunt, Catheter, Cerebrospinal fluid (CSF), Obstruction, Soft microdevices

## Abstract

Ventriculoperitoneal (VP) shunt obstruction, often caused by protein and fat accumulation at the ventricular catheter ports, impedes cerebrospinal fluid (CSF) outflow, increases intracranial pressure (ICP), and leads to hydrocephalus. Current treatments require invasive shunt removal, reimplantation, or retrograde flush cleansing. We present a next-generation VP shunt system that actively removes blockages via external actuation. Our system, called CLogging Elimination ActuatoR Silicone (CLEARS), integrates a soft, expandable silicone tube within the catheter lumen. This soft robotic insert, capable of 900% strain, can inflate to dislodge blockages and then deflate to restore flow. To test CLEARS, we developed an ex vivo model simulating CSF flow and obstruction using a rapidly acting clogging agent. ICP upstream of the catheter was monitored to evaluate performance. When obstructed with 3 g of the clogging agent, ICP rose to 30 cmH$_2$O. Upon CLEARS activation, the silicone insert expanded through catheter ports and successfully removed the clog, restoring baseline ICP ($\sim$0 cmH$_2$O) within approximately 40 s. Without the system, obstruction persisted and pressure remained elevated. Visual documentation confirmed the mechanism of action. The CLEARS system offers a promising solution to VP shunt occlusion by enabling non-invasive mechanical declogging. Our model replicates shunt obstruction and CSF dynamics, providing a testbed for device evaluation. The expandable insert maintained catheter flow and reduced ICP to normal levels after obstruction, representing a potential shift in how hydrocephalus is treated.

**Supplementary Information:**

The online version contains supplementary material available at 10.1007/s10544-025-00769-8.

## Introduction

Hydrocephalus is a neurological disorder caused by excess cerebrospinal fluid (CSF) within the brain’s ventricular system. The abnormal buildup of CSF, an incompressible Newtonian fluid with a density and viscosity similar to water (Balasundaram et al. 2023), enlarges the ventricles, compressing neural pathways and increasing intracranial pressure (ICP). The CSF is partly secreted by the choroid plexus (ChP) (Ueno et al. 2024); 25 mL of it is present in the brain’s ventricles. The ChP, a layer of epithelial tissue on the ventricle wall, regulates CSF secretion. CSF maintains ionic homeostasis, absorbs shocks, and preserves blood-brain barrier integrity (Ueno et al. 2024). It consists of proteins, albumin, vitamins, lipids, and essential ions for neural function (Spector et al. 2015).

Patients with hydrocephalus often experience headaches, impaired coordination, and blurred vision. Untreated hydrocephalus can rapidly progress to cognitive deficits or death (Khazim et al. 2015). Hydrocephalus can be caused by many conditions, but most often occurs from infection (Kim et al. 2006) or the abnormal resorption of CSF following stroke or traumatic brain injury (Edwards et al. 2004). With over 400,000 cases annually worldwide (Dewan et al. 2018), untreated hydrocephalus in pediatric patients can hinder mental development and produce learning disabilities (Szefczyk-Polowczyk and Mandera 2020).

The most common treatment of hydrocephalus is the implantation of a ventriculoperitoneal (VP) shunt. The VP shunt has three components. The ventricular catheter is a small tube with perforations at the distal end and is designed to reside in the lateral ventricles of the brain to allow CSF to flow out of the intracranial space. A valve is attached to the catheter under the scalp to regulate CSF flow along the central lumen of the catheter. A distal catheter attached to the valve is surgically tunneled under the skin and terminates in the peritoneal cavity of the abdomen, where excess CSF is deposited. About 36,000 shunts are implanted each year (Bondurant and Jimenez 1995).

Unfortunately, VP shunts fail in up to 85% of cases within 10 years, most often due to obstruction of the ventricular catheter (Garcia-Bonilla et al. 2024). Obstructions are typically caused by protein buildup or choroid plexus tissue ingrowth (Levrini et al. 2020). These failures often require revision surgeries; in 2005, half of the $1B U.S. expenditure on shunt procedures was for revision surgeries alone (Nabbanja et al. 2018). Gupta et al. (2007) found that 54% of shunt patients required at least four revisions, with infection rates between 5% and 15% within the first month (Choux et al. 1992). Despite over 50 years of medical advances, the revision rate has not improved (Kofoed Månsson et al. 2017). Shunt obstructions are considered a medical emergency (Ferras et al. 2020) and can rapidly result in serious consequences, particularly an increase in ICP. This can trigger altered mental states, bradycardia, vomiting, infection, or other symptoms associated with hydrocephalus. Surgical intervention is necessary and typically requires hospitalization. A common intervention is a retrograde flush into the shunt that is used to remove the occlusion (Nabbanja et al. 2018).

Recent efforts to improve VP shunts have explored both passive and active methods to reduce clogging. Galarza et al. (2018) used finite element analysis to optimize shunt perforations, and such passive methods still leave a significant risk of clogging. Lutz et al. (2013) proposed the development of a “smart shunt” by integrating sensors capable of transmitting real-time shunt status to mobile devices, which will notify the patient of a clog before physiological and psychological effects become apparent, saving precious time in treatment. Newer shunts employ a combination of silicone and nitinol instead of plastic alone (Emery et al. 2023); these shunts can change their size due to the property of nitinol wire while the silicone controls the initial geometry of the shunt (Gilbert and Webster 2015; Nandasiri et al. 2020), though these appear to clog as easily as the earlier designs. Sensing the onset of an obstruction in a shunt catheter is beneficial as well, and work by Hudson et al. (2021) demonstrated precise measurement of the flow of CSF from the brain via a shunt.

Noting a new generation of neurosurgical treatment devices beginning to appear (Ullah et al. 2025; Singh et al. 2025), our objective is to deliver a next-generation shunt system—the CLogging Elimination ActuatoR Silicone (CLEARS) system—with the ability to *safely* eliminate obstruction. The remainder of the text describes the design of the CLEARS system, the development of a rapidly clogging material useful in an *ex vivo* experiment, and the experiment itself, incorporating a fluid flow system design that appropriately models the intracranial pressure increase that occurs upon clogging of the shunt. Results from this approach are then provided, with a comparison of a standard shunt with one that incorporates the CLEARS system.

**Fig. 1:**
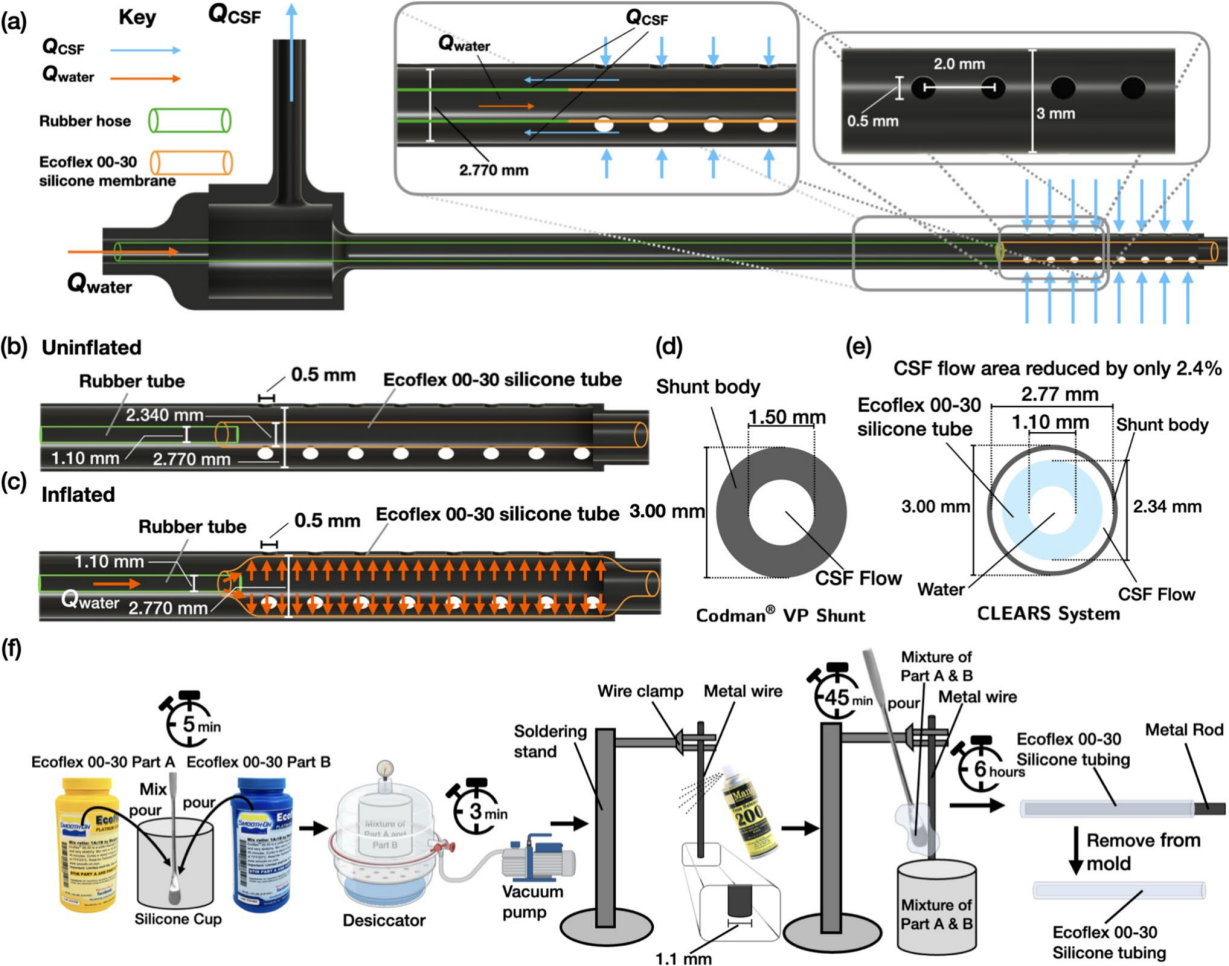
(**a**) An illustration of the next-generation VP shunt with CLEARS system, with (**b**) the uninflated silicone membrane installed into the ventricular catheter, and (**c**) after inflation using saline or water. Water was used in our experiments while saline would be used in the *in vivo* application. (**d**) A cross-sectional view of the Codman^®^ ventricular catheter, indicating the region where CSF may flow in white. (**e**) A cross-sectional view of the CLEARS system which has a reduced shunt wall thickness with silicone tubing (sky blue) inside. The silicone tubing has an outer diameter of 2.34 mm. (**f**) The fabrication procedure for the Silicone membrane. The silicone is produced by thoroughly mixing 5.0 g of part A and 5.0 g of part B (as a two-part composition per instructions for Ecoflex 00–30) in a silicone cup and placing the mixture in a vacuum desiccator for approximately 3 min to remove dissolved air from the mixture. A rod (1.1 mm diameter circular metal rod, uxcell, Beijing, China) serves as a mold, held vertically with a simple soldering stand. Mold release was twice applied to the rod, cured, and then the silicone was repeatedly applied using a spatula over a 45 min period. Flowing downward under gravity, the silicone uniformly coated the rod with a thickness dependent upon its viscosity. The entire setup was left untouched for 6 h to ensure complete curing

## Methods

### Next-generation VP shunt with an integrated soft robotic device

We developed a modified ventricular catheter incorporating a soft, expandable silicone insert inspired by steerable neurointerventional microcatheters (Gopesh et al. 2021). We introduce a radially expandable tube within the inner lumen of the ventricular catheter. The inserted tube remains collapsed during normal flow and expands radially when inflated with saline or water, displacing clogging materials through the catheter’s ports. Parts of the expandable silicone insert protrudes through the shunt ports to displace clogging material (Fig. 1(b,c)). Upon deflation, the ports and lumen reopen, resuming CSF flow.

In this prototype, the size of the catheter’s lumen was slightly increased to maintain a nearly identical cross-sectional area for CSF flow to the original catheter design. We increased the inner diameter of the ventricular catheter lumen from the Codman^®^ shunt from 1.5 mm to 2.77 mm, maintaining the standard outer diameter of 3.0 mm (see Fig. 1(d,e)). Our custom designed catheter body was resin printed using a biocompatible material (BioMed Durable Resin, Formlabs Inc., Somerville, MA USA). This allowed space for a custom-fabricated silicone tube (Ecoflex 00–30, Smooth-On, Macungie, PA USA) with an inner diameter of 1100 $\mu$m and outer diameter of 2340 $\mu$m. The soft insert was fabricated from Ecoflex 00–30 silicone (Ecoflex 00–30, Smooth-On, Macungie, PA USA; maximum strain: 900%), using a process similar to Fan et al. (2023), a biocompatible material (Lavazza et al. 2023) with an inner diameter of 1100 $\mu$m and outer diameter of 2340 $\mu$m, reducing the cross-sectional area by only 2.3% (Fig. 1(e)). The expandable insert was connected to a 3 mL syringe (BD 3ml Syringe Luer-Lok$^{\text {TM}}$ Tip, Becton, Dickinson and Company, New Jersey, USA) via a rigid 1.0 mm outer, 0.5 mm inner diameter silicone tube (CGjiogujio, Nanjing, China). Because saline and water possess no mechanical differences at these scales, water was used as the working fluid. The silicone portion inside the shunt deflated rapidly upon syringe pressure release. A force of $1.69\pm 0.1$ N is needed to inflate our device, much less than the approximate 100 N grip force threshold exhibited by 50$^\text {th}$ percentile humans (Wang et al. 2018). Over time, it is reasonable to expect this inflation force to decrease due to the Mullins effect (Krpovic et al. 2021), a fatigue-driven effect that can be compensated for.

The inflatable silicone tubing was fabricated via a process illustrated in Figure 1(f). Fabrication involved coating a 1.1-mm diameter stainless steel mandrel with mold release, casting the silicone, curing at 25°C for 6–8 hours, and sealing one end. The tubing was peeled from the mandrel and cut to the desired 25 mm length, corresponding to the length of the portion of the shunt with ports. It was then cleaned using a small amount of isopropyl alcohol (IPA), followed by deionized water, and sealed at one end with a second application of Ecoflex 00–30 to 1.0 mm in length along the inner lumen of the Ecoflex tube. The insert was attached to non-inflating tubing using cyanoacrylate glue (CA-MG InfinityBond, Santa Monica, CA USA) and epoxy (Adhesive 81, Norland Inc., Jamesburg, NJ USA), cured with a 365 nm ultraviolet light (Blacklight UV301D, Lightfe, Shenzhen, China). The total length matched the catheter’s ported region (25 mm). It may be necessary to consider surface treatment of the silicone material, such as using polyvinyl acetate coatings (Kim et al. 2022), to protect the expandable material for its long-term use in the brain.

### Clogging agent

Existing models from the literature use egg components including vitelline and shell membrane as clogging agents for ventricular catheter obstruction but produce limited volumes (Qi et al. 2023; Flürenbrock et al. 2023). Each egg contains only a small volume of vitelline and shell membrane compared to the rest of the egg. In our model, egg albumin, shell membrane, and chalaza were first harvested from the egg. Larsen and Froning (1981) suggested utilizing IPA to solidify egg yolk for use as a clogging agent. The vitelline was first punctured to allow the outflow of the egg yolk, and the egg yolk and vitelline were then separated via mixing with $99\%$ IPA. The egg albumin was solidified via heating at $T=115-130^{\circ }\textrm{C}$ for about 3 min or until sufficient solidified albumin existed, and then the materials were mixed to produce the final clogging agent.

Approximately 3.0 g was added to the model ventricle before each trial. The occlusion of the shunt was achieved by vigorous shaking. This clogging agent was used in all the shunt assessments with clogging reported in our study. Our approach produces clogs in seconds, ideal for proof-of-concept experiments to test the declogging of these catheters.

### Model of intracranial pressure and installation of ventricular catheter

**Fig. 2:**
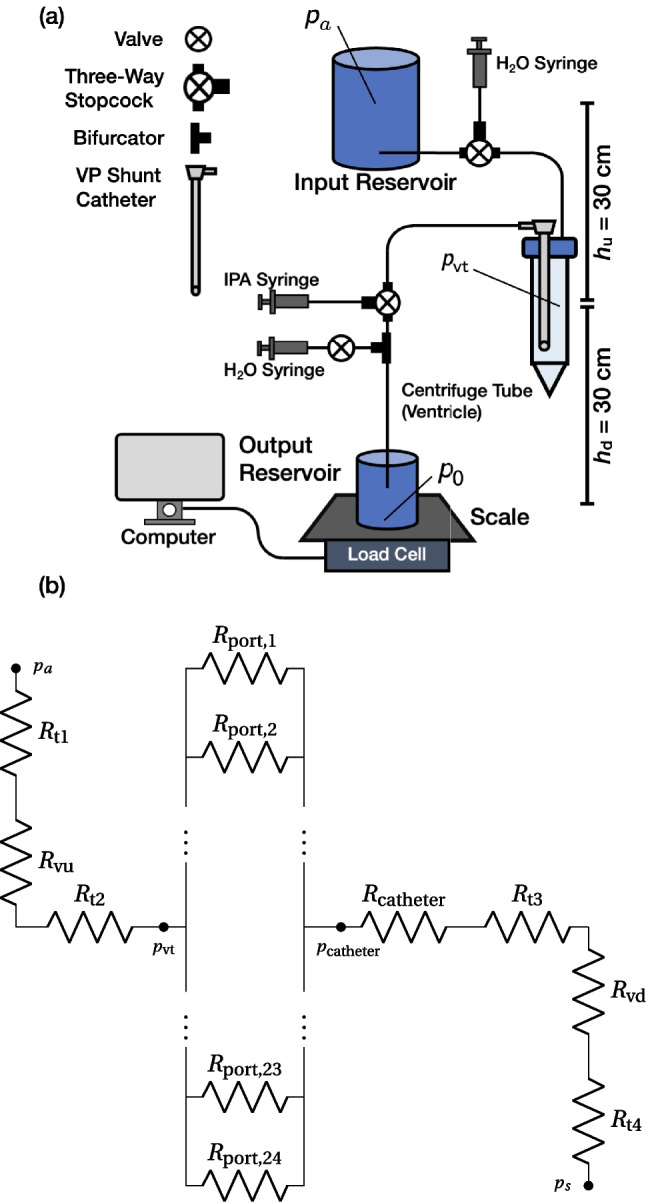
(**a**) A schematic of the fluidic circuit representing the shunt system and patient. The input reservoir is $h_u=30$ cm above the centrifuge tube. The tube represents the ventricle; note the ventricular catheter present within it. The output reservoir is $h_d=30$ cm below the centrifuge tube. The fluid flows from the input reservoir to the output reservoir via the centrifuge tube and shunt. The IPA and $\text {H}_2\text {O}$ syringes initially fill the circuit to eliminate trapped air before being switched off to allow flow solely from the input reservoir. The mass of the output reservoir is measured and recorded every 5 s via the load cell. (**b**) A simple circuit representation of the setup may be used to determine the pressures present in the system with knowledge of the flow rate measured from the change in the output reservoir mass by the load cell. See text for component definitions

Past models require stopping the flow to measure the clogging of the catheter ports (Qi et al. 2023), and lack the ability to continuously operate through a clogging event. We instead designed a gravity-driven fluidic circuit using water as a CSF analog as shown in Fig. 2, incorporating the ability to clog the implanted shunt and monitor the consequent increase in CSF pressure in our model ventricle.

To evaluate our CLEARS system, we compared an existing ventricular catheter from Codman^®^ to a ventricular catheter with our system. We excluded a valve from the system to simplify the fluid circuit, focusing on the ventricular catheter, justified by the clinical use of ventricular catheters without valves, known as external ventricular drains (EVDs) (Muralidharan 2015). The occlusion rates of EVDs are similar to or higher than permanently implanted catheters, with one study reporting a 42% occlusion rate over 10.9 days (Fargen et al. 2016). Commercial valves are not ubiquitous and include subcomponents such as pump reservoirs and anti-siphon devices, making their behavior complex.

A 50 mL centrifuge tube simulated the brain ventricle in this system. Water flowed from a reservoir placed 30 cm above the simulated ventricle, into that ventricle, through the catheter, and onwards to a second reservoir placed 30 cm below the simulated ventricle. Pressures were defined by the relative height of the reservoirs and centrifuge tube. The clogging agent was introduced into the artificial ventricle before the trials. The output reservoir’s mass was measured at 5 s intervals using a load cell (HX711, Avia Semiconductor, Xiamen, P. R. China) connected to a computer via Arduino. Pressure in the centrifuge tube was determined by component resistances (Fig. 2(b)) and the flow rate from the reservoir’s mass.

We further constructed a simple model of the system using an electrical circuit analog, as shown in Fig. 2. The resistance of the silicone tubes connecting the reservoirs and the simulated ventricle is denoted by $R_{\textrm{t1}}$, $R_\textrm{t2}$, $R_\textrm{t3}$, and $R_\textrm{t4}$. The ventricular catheter’s 24 ports’ resistances are $R_{\textrm{port,i}}$ where $i=\{1,2,3,...,23,24\}$, while $R_{\textrm{catheter}}$ is the resistance from the ventricular catheter internal lumen. The valves in our experimental system also exhibit unknown fluidic resistance and are represented by $R_{\textrm{vu}}$ and $R_{\textrm{vd}}$. They were determined by calibration with the experiment, as all other component resistances could be calculated.

With the pressure at the upper reservoir defined as $p_a$, the ICP $p_{\text {vt}}$ in Fig. 2 is given by

1 $$\begin{aligned} p_{\textrm{vt}}(t_i)=p_a-Q\left( t_i\right) R_{u}, \end{aligned}$$

where $R_u$ is the resistance of the fluidic circuit upstream of the ventricle and $Q\left( t_i\right)$ is the flow rate. The experimental flow rate $Q(t_i)$ was computed using the Euler forward finite difference approximation for the time derivative,

2 $$\begin{aligned} Q\left( t_i\right) =\frac{1}{\rho \Delta t}\left( m(t_i+\Delta t)-m(t_i)\right) , \end{aligned}$$

where $\rho$ denotes the density of water and $m(t_i)$ the mass in the output reservoir. Converting the experimental setup in Fig. 2(a) into the circuit shown in Fig. 2(b) produces the upstream resistance,

3 $$\begin{aligned} R_{u} = R_{\textrm{t1}}+R_{\textrm{t2}}+R_{\textrm{vu}}. \end{aligned}$$

The water flow in this circuit may be assumed to be Poiseuille flow, producing a flow rate, *Q*, which depends upon the pressure difference $\Delta p$ from input to output,

4 $$\begin{aligned} Q=\left( \frac{A^2}{8\pi \mu L}\right) \Delta p, \end{aligned}$$

where *A*, *L*, and $\mu$ denote the cross-sectional area and length of the tubing and the dynamic viscosity ($8.90\times 10^{4}$ Pa$\cdot$s) of the fluid, respectively. Using Eq. 4 and the analogous Ohm’s law, $Q=\Delta p/R$, each of the resistances in Eq. 3—except for $R_{\textrm{vu}}$—are given by

5 $$\begin{aligned} R=\frac{8\pi \mu L}{A^2}. \end{aligned}$$

The value of $R_{\textrm{vu}}$ is found by comparing the results of the simple analysis model and the experiment. The experimental flow rate was slightly slower, and this was assumed to be due to resistance in the valves. Using Eqs. 1–3, the intracranial pressure $p_{\textrm{vt}}$ at time $t_i$ is

6 $$\begin{aligned} p_{\textrm{vt}}(t_i) = \gamma h_u-\frac{R_u}{\rho \Delta t}\left( m(t_i+\Delta t)-m(t_i)\right) , \end{aligned}$$

where $\gamma$ is the specific weight $\left( 9.807~\mathrm {kN\cdot m}^{-3}\right)$ of water.

**Fig. 3:**
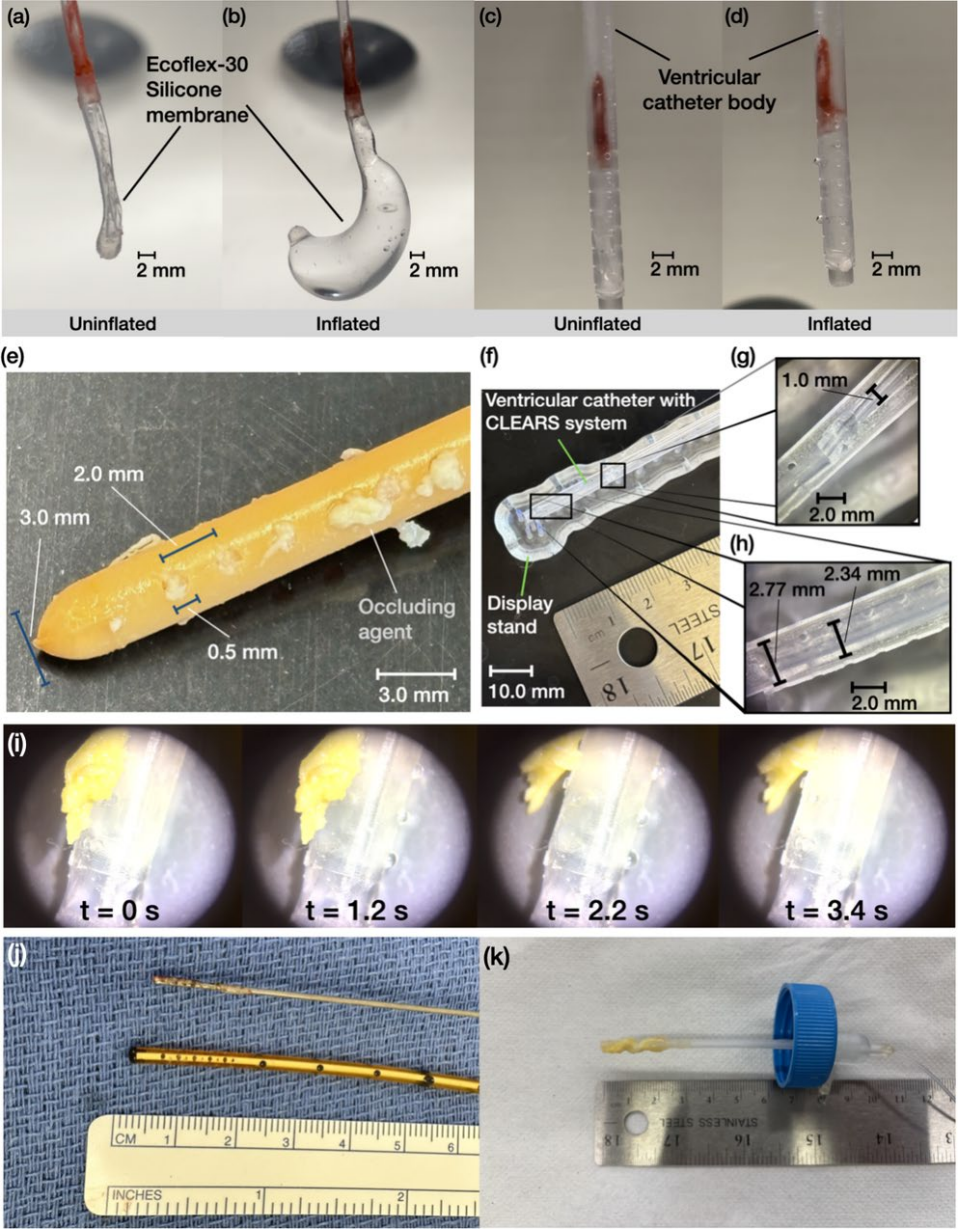
(**a**) Uninflated silicone insert. (**b**) As water is pumped into the silicone membrane from hand pressure applied to a connected syringe, the silicone membrane inflates. Placed (**c**) into a transparent version of the shunt (for visibility), the silicone structure allows passage of fluid inside when uninflated. When (**d**) inflated, the silicone structure expands against the inner lumen of the shunt, and portions of the silicone insert (*) exude from the pores of the shunt. All scale bars are 2 mm. (**e**) The Codman^®^ ventricular catheter with the clogging agent clogging the ports of the shunt after 200 s exposure to the clogging media. (**f**) The cross-sectional view of the next-generation ventricular catheter with the CLEARS system. (**g**) A close-up image of the juncture between the expandable, soft silicone portion and the attached, more rigid silicone tubing supplying a working fluid, all within the ventricular catheter. (**h**) A close-up image of a portion of the CLEARS soft silicone device inside the ventricular catheter with ports. (**i**) Unclogging using the CLEARS system over 3.4 s. Water is pumped into the soft silicone portion of the CLEARS to radially expand it over the first 1.2 s. The clogging material is pushed out of the ventricular catheter’s ports from 1.2 s to 2.2 s. At 2.0 s, the hand pressure used to expand the CLEARS device is released, and the device deflates to its original shape by 3.4 s. A video of the unclogging process is provided for this paper. (**j**) An inner rigid stylus withdrawn from a Codman^®^ proximal shunt catheter shows proteinaceous debris clogging it. (**k**) The clogging agent obstructs the CLEARS system-based shunt if the clearing mechanism is not activated

## Results

The uninflated and inflated states of the silicone insert for the CLEARS device, both inside and outside the catheter, are shown in Fig. 3(a-d). Injecting water into the proximal end of the device inflates the silicone portion. When in the shunt, the silicone membrane expands and pushes against the interior wall of the shunt body, and those portions of the membrane at the pores exude out of the pores and appear on the outside of the shunt, as shown in Fig. 3(d). In Figure 3(e), a Codman^® ^shunt is shown clogged with our test material while in the fluid circuit.

The CLEARS device as fabricated is shown in Fig. 3(f-h), and as clogged—and then unclogged—in our experimental setup in Fig. 3(i) and Supplementary Video 1. The clogging agent was first introduced into the ventricular catheter’s ports, using typical CSF flow conditions to mimic how an actual ventricular catheter obstruction occurs, clogging the ports at $t=0$ s. The CLEARS system was activated at $t=1.2$ s, and the inflation occurred at $t=1.4$ s, taking 0.2 s to pass sufficient fluid into the expandable silicone insert to inflate it. From $t=1.4$ s to $t=2.2$ s, the CLEARS system expanded and pushed through the catheter’s ports to dislodge the clogging agent at $t=2.2$ s. Releasing the hand pressure from the syringe used to drive the water into the CLEARS system at $t=2.2$ s, the device deflated and left the clogged material separated from the ports. The clogging appears qualitatively similar between a ventricular catheter extracted from a patient (Fig. 3(j)) and the CLEARS device after use in our setup (Fig. 3(k)).

Four trials were conducted using the ICP ($p_{\text {vt}}$ in Fig. 2(a,b)), Codman^®^ ventricular catheter with and without clog (Fig. 2(a)) and the CLEARS catheter with and without clog (Fig. 2(b)). The ICP remained at 0 cmH$_2$O without clogging in both devices. After the clog was introduced at 20 s in the Codman^®^ device and 105 s in the CLEARS device, the ICP began to rise to 30 cmH$_2$O over about 100 s, indicating full obstruction. CLEARS activation at $t \approx 485$ s caused rapid inflation and unclogging from $t \approx 525$ s. Following device deflation, ICP dropped to 0 cmH$_2$O. A second activation at $t \approx 545$ s, ending at $t \approx 565$ s, confirmed consistent performance. After unclogging, the ICP stabilized at pre-obstruction levels, demonstrating full restoration of catheter function.

**Fig. 4:**
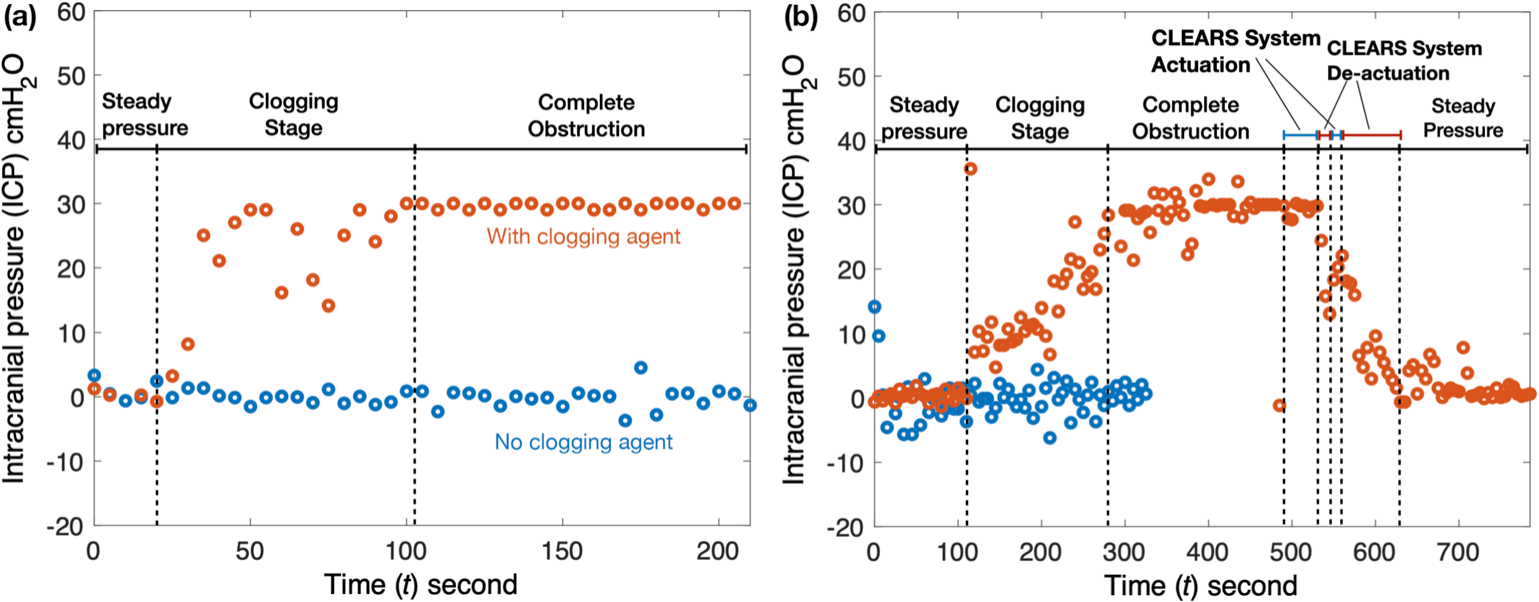
The intracranial pressure (ICP) for both the Codman^®^ shunt and a VP shunt with CLEARS device evaluated in a fluidic circuit (Fig. 2) without a shunt valve. (**a**) The intracranial pressure is plotted versus time for a Codman^®^ shunt without (blue) and with (orange) a clogging agent. Obstruction of the shunt begins at about 20 s and is completely clogged at about 110 s. (**b**) Shows the intracranial pressure for the CLEARS system without a clogging agent (blue) and the other one with a clogging agent (orange). Pores obstruction begins at 105 s and complete obstruction occurs at 280 s. First CLEARS actuation occurs at $t\approx 485$ s and deactuated at $t\approx 525$ s. The second CLEARS actuation occurs at $t\approx 545$ s and de-actuates at $t\approx 565$ s

## Discussion

Ventriculoperitoneal (VP) shunts remain the standard treatment for hydrocephalus (Javeed et al. 2023), yet obstruction is common, affecting patients’ quality of life, (Garton et al. 2001), requiring urgent intervention (Ferras et al. 2020) and contributing to high revision rates and healthcare costs (Bondurant and Jimenez 1995). The CLEARS system offers a mechanical, non-invasive approach to restore flow and reduce intracranial pressure (ICP).

Our modified catheter, incorporating a radially expandable silicone insert, successfully restored baseline ICP after clogging. Without CLEARS, obstruction raised ICP to 30 cmH$_2$O, surpassing the clinical threshold for hydrocephalus (Lee and Lueck 2014). Activation of the CLEARS system rapidly reduced ICP to 0 cmH$_2$O, demonstrating effective unclogging. Minor noise in ICP results, likely due to load cell sensitivity, was observed. As it is, the technology is a demonstrator of the potential to disrupt obstructions that form at the cerebral catheter pores, where the vast majority of flow obstructions occur in these systems (Paff et al. 2018). However, the risk of obstructions in the peritoneum is also present, typically due to infection or foreign bodies—cotton, hair, or tissue—introduced during placement of the shunt (Sekhar et al. 1982). It is not entirely clear what the long-term fate of disrupted catheter pore obstructions will be, because the typical practice is to replace the catheter in an emergent response. It may be possible to pharmacologically dissolve or reduce this debris over a longer period, an action that would be facilitated by immediate treatment of the obstruction through the use of CLEARS or similar technology. Past studies (Del Bigio and Di Curzio 2016) indicate that acetazolamide bolus may be effective for this purpose, but the evidence is preliminary.

Our focus is the minimally-invasive disruption of obstructions in the cerebral catheter, and we envisage a version of this technology that will employ the programmable valve body present in modern versions of the shunt to incorporate a small fluidic pump (Bußmann et al. 2021) appropriate to inflate the CLEARS device, based upon pressure and flow changes detected at the valve. Because it can use sterile saline as a working fluid in an enclosed system, the risk of problems with the technology are reduced. While the proposed CLEARS mechanism does introduce working fluid into an enclosed brain-adjacent system, producing a possible infection risk, by using sterilized CSF analog and durable elastic media with a maximum strain exceeding 900%, we minimize this risk to a level we anticipate to be lower than the risk of clogging of traditional shunts.

The device was manually fabricated in a process that is certainly subject to variability. Borne of necessity, it was sufficient for our demonstration purposes in this study, but the appropriate way to do this for repeatability, certainly in the actual application itself, would be to cast mold the devices. We anticipate doing this in future work, as a part of an overall process appropriate for medical devices with design controls and adherence to quality standards (Medina et al. 2013; Zenios et al. 2010). An important part of this process is the selection and modification of the soft silicone material used to form the CLEARS device. Silicone has been modified (Li et al. 2012) to be biocompatible (Williams 2008) in many applications, including the brain (Kuddannaya et al. 2015; Kumosa 2023), and we anticipate this will be possible here. These aspects are beyond the research focus of this work but certainly necessary for development of the technology.

Our *ex vivo* model represents ventricular catheter use with and without clogging. The model’s advantages over the current state of the art include a realistic ICP increase during CSF generation and the induction of catheter obstruction by manual shaking of the catheter-containing vial. This improves upon prior models that required disassembly (Qi et al. 2023). Our setup also produced constant ICP with functioning catheters, as shown in Fig. 4(a,c); past approaches have only produced transient changes in the ICP that were implied to represent the much slower changes in ICP that occurs in patients. Our clogging agent mimicked the proteinaceous, cellular debris found in clinical cases (Hanak et al. 2016), as seen in prior work where small portions of chicken egg—the vitelline or chalaza—were used for this purpose (Qi et al. 2023; Flürenbrock et al. 2023). We were able to generate much larger quantities of clogging agent by using the entire egg white. Our model also provided relatively precise measurement of the flow and pressures in the system, important because both are quite small, and because minor changes in these values are clinically important.

The model’s disadvantages include the fact the clogging is driven suddenly into place, unlike actual VP shunt obstruction which tends to occur over weeks to years. This is intrinsically a challenge in any experimental model where one cannot wait for the clog to naturally develop over such a long period of time. The proposed clogging agent resembled clogs collected from patient shunts and produced similar morphologies (*see* Fig. 3(e,j,k)). The model also omits valves present in actual use (Reis et al. 2023), resulting in a measured ICP of 0 cm H$_2$O. Including a valve would increase resistance, decrease flow rate, and raise ICP $(\textrm{ICP}>0$ cm H$_2$O), per Eq. 6, commensurate with its clinical use. Our model omits the peristalsis in ICP flow that is known to exist due to the cardiac cycle driving blood vessel motion (Mestre et al. 2018), is physiologically important, and may impact clogging of the shunt over the long term. Finally, our proof-of-concept model produces clogs when the experimenter shakes the vessel containing the clogging material and the shunt, making replicates with similar timing for the clogging event very difficult to achieve. Future improvement in the model should incorporate a method for automatically delivering the clogging material on command.

## Conclusions

We present a next-generation ventricular catheter system integrated with a soft robotic device, evaluated in a fluid circuit model using an improved clogging agent. Intracranial pressure was the key outcome measure. This ventricular catheter restores steady-state pressure equivalent to normal intracranial levels shortly after a clogging event, achieved through the expansion of an internal silicone component that protrudes through the catheter ports to physically dislodge and remove occlusive materials.

## Supplementary Information

Below is the link to the electronic supplementary material.

Supplementary file 1 (zip 23842 KB)

## Data Availability

No datasets were generated or analysed during the current study.
