# Supplementary figures and images for: Uncloggable ventriculoperitoneal shunt system for hydrocephalus via an integrated soft robotic device: CLEARS device
Source: Biomed Microdevices. 2025 Sep 11;27(3):41. doi: 10.1007/s10544-025-00769-8 (PMC12426090; doi:10.1007/s10544-025-00769-8)

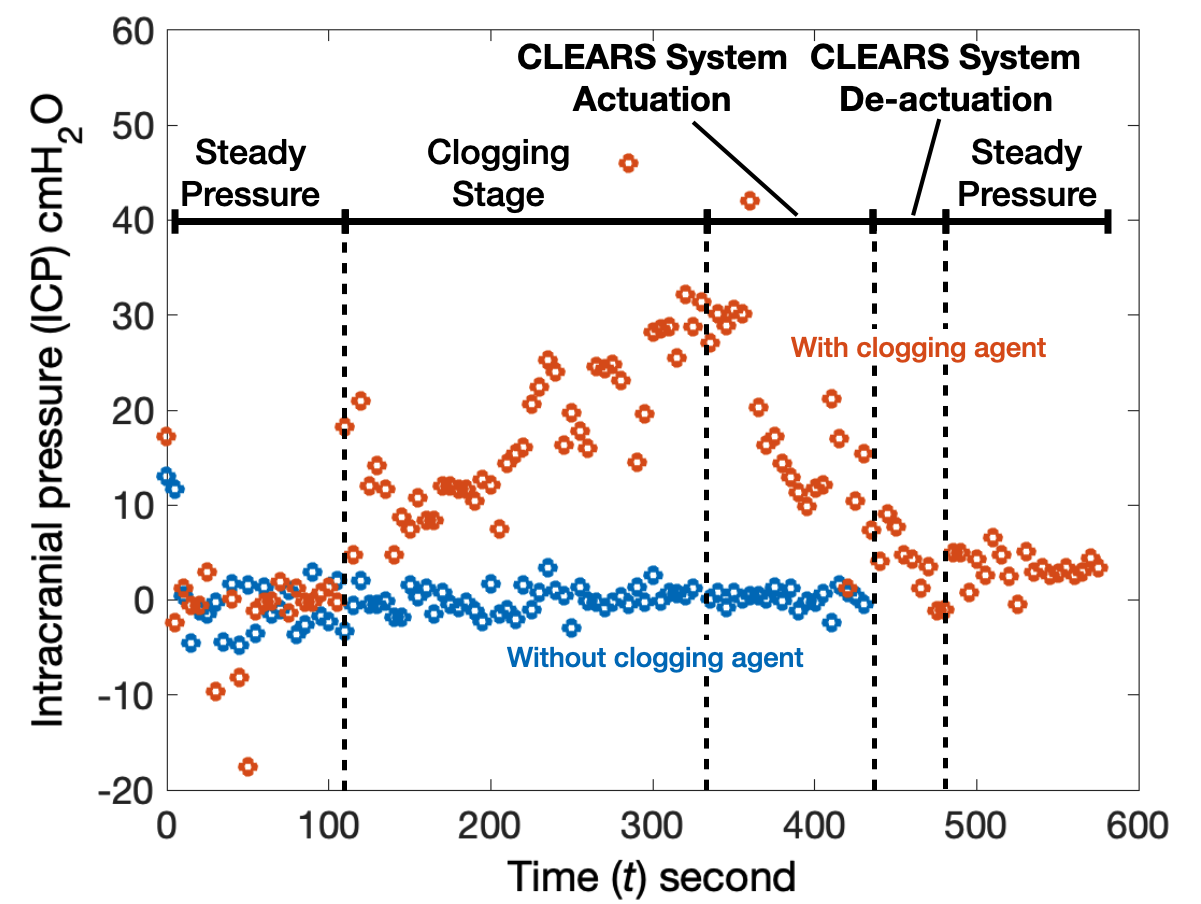

Supplement: Supplementary file 1 — (zip 23842 KB) [file 10544_2025_769_MOESM1_ESM.zip › SupplementaryFigure1-ICPvsTimewithdeclogging.png]
